# Human Infection with *Cryptosporidium felis*: Case Report and Literature Review

**DOI:** 10.3201/eid0801.010269

**Published:** 2002-01

**Authors:** Simone Cacciò, Elena Pinter, Rosanna Fantini, Ivano Mezzaroma, Edoardo Pozio

**Affiliations:** * Istituto Superiore di Sanità, Rome, Italy; † University of Rome La Sapienza, Rome, Italy

**Keywords:** *Cryptosporidium felis*, HIV, paromomycin, treatment, CD4+ cell, erythrocine

## Abstract

An infection with *Cryptosporidium felis* in an HIV-positive man from Italy was successfully treated with paromomycin, despite the patient’s having a CD4+ cell count of 31/mL. Fourteen cases of human infection with *C. felis* have been described, all in the past 3 years, emphasizing the public health importance of *Cryptosporidium* parasites other than *C. parvum*.

Parasites belonging to the genus *Cryptosporidium* are an important and widespread cause of enteric disease in humans and in many other vertebrates. The most commonly identified etiologic agent of human cryptosporidiosis is *Cryptosporidium parvum* (*1*), which, based on the molecular characterization of oocysts, can be divided into two genetically distinct subpopulations: genotype 1 (or the “anthroponotic genotype”), which is associated exclusively with human infection; and genotype 2 (the “zoonotic genotype”), which is associated with both human and animal infection (2). For many years, *C. parvum* was considered to be the only etiologic agent of human infection. However, the use of molecular tools with a greater capacity to detect and differentiate strains has resulted in the identification of other human pathogens: *C. felis*, *C. meleagridis*, the *C. parvum* dog genotype, and possibly *C. muris* (*2*).

In Italy, all *Cryptosporidium* oocysts detected in immunocompetent and immunosuppressed persons have been identified as *C. parvum* (*3*). In this report, we describe the first Italian case of a human *C. felis* infection, which occurred in an HIV-positive man who, in spite of a very low CD4+ cell count, successfully recovered with paromomycin treatment.

## The Study

On December 12, 2000, a 42-year-old homosexual man with severe diarrhea was admitted as an outpatient to the Umberto I° University Medical Center in Rome, Italy. The man had been diagnosed with *Hepatitis B virus* infection in 1982 and with HIV infection in 1988. In 1989, his CD4+ cell count had reached 189/mm^3^, and he started antiretroviral therapy with zidovudine. He was later treated with all nucleoside analogs (individually or combined), and in 1996 he began treatment with highly active antiretroviral therapy (HAART). The specific HAART regimen was changed several times because of his poor immunologic and virologic response. In the course of years, the man had many episodes of oral candidiasis, which were responsive to fluconazole therapy. In July 2000, he weighed 87 kg and had a CD4+cell count of 52/mm^3^; at that time, HAART was changed to include ritonavir, indinavir, stavudine, and didanosine. In November 2000, the patient had isolated episodes of diarrhea, but feces were not investigated for the presence of parasites. At the beginning of December 2000, he was affected by impetigo, which was treated with erythrocine (1800 mg/day). Two days after beginning that therapy, he began to have up to 10 episodes of diarrhea per day. Erythrocine was interrupted after 3 days, and, in the period of 1 week, the patient lost 9 kg. Stool specimens were tested for a wide panel of enteric pathogens (bacteria, viruses, helminths, and protozoa, including microsporidia). The parasitologic examination of stools showed *Cryptosporidium* oocysts (3x10^6^ oocysts/mL of feces). The oocyst diameter was in the range of 4.5-4.9 _m. Oocysts reacted strongly with two monoclonal antibodies conjugated with fluorescein (MeriFluor, Meridian Diagnostics, Cincinnati, OH; *Cryptosporidium* Immunofluorescence Test, Microgen Bioproducts Ltd, UK). No other pathogen was found in the specimens. The patient was treated with paromomycin (1 g, 3 times/day). On the second day of treatment, the diarrhea promptly resolved, decreasing from 10 to 2 bouts per day. Paromomycin treatment was continued until mid-February (CD4+ cell count 31/mm^3^) without further diarrheal episodes, and stools were negative for *Cryptosporidium*.

DNA was extracted from the whole feces according to the FastPrep method of da Silva et al. (*4*), and the diagnostic fragment of the small subunit ribosomal RNA (ssu-rRNA) was amplified by polymerase chain reaction (PCR) with the primer set CPBDIAGF and CPBDIAGR (*5*). The sequence of the PCR product was determined, and a comparison with all ssu-rRNA *Cryptosporidium* sequences available in databanks revealed 100% similarity with the homologous fragment of *C. felis* (accession number AF087577). To obtain additional information on the nature of the species, a PCR-restriction fragment length polymorphism assay (primer set cry9 and cry15) (*6*) that targets a fragment of a *Cryptosporidium* oocyst wall protein gene was used (*7*). This second analysis confirmed the identification of *C. felis* (Figure).

**Figure:**
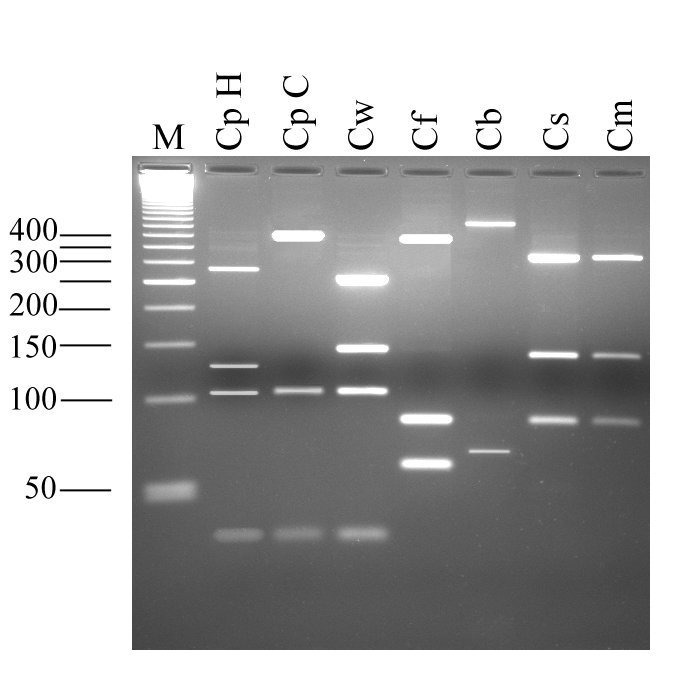
Electrophoretic separation of *Cryptosporidium* oocyst wall protein gene-polymerase chain reaction products digested with the endonuclease *RsaI*. Lane M, 50-bp size ladder; CpH, *Cryptosporidium* *parvum* human genotype; CpC *C. parvum* calf genotype; Cw, *C. wrairi*; Cf, *C. felis*; Cb, *C. baileyi*; Cs, *C. serpentis*; Cm, *C. muris*.

To the best of our knowledge, this is the only case of a *C. felis* infection for which the clinical course and the response to therapy have been reported. Although the literature contains numerous reports of paromomycin treatment of human *Cryptosporidium* infection, the results regarding the efficacy of paromomycin are contrasting, possibly because it was always assumed that the etiologic agent was *C. parvum*. The current knowledge that several species and genotypes can infect humans suggests that the efficacy of paromomycin could depend on the specific *Cryptosporidium* species/genotype and its susceptibility to this drug. In our case report, the infected person had a very low CD4+ count, which has been considered as one of the most important factors in the failure of paromomycin treatment (*8*,*9*). The concomitance of the erythrocine treatment and severe watery diarrhea suggests that the drug had altered the intestinal flora and, in turn, favored the growth of the parasite. A concomitant influence of paromomycin treatment and the interruption of erythrocine treatment can be also postulated.

There have been 14 cases of human infections with *C. felis* reported; all have occurred in the past 3 years (*10*–*13*). These cases occurred in North and South America, Africa, and Europe, and they involved both immunocompetent (n=4) and immunosuppressed (n=10) persons.

There have also been cases of human cryptosporidiosis in which cats were identified as the source for human infection, yet the species of *Cryptosporidium* remained unknown. Glaser et al. (*14*) examined the association between *Cryptosporidium* infection and animal exposure in HIV-infected persons and concluded that only dog ownership presents a risk, although minimal; no significant risk was associated with cat ownership. However, cats have been successfully experimentally infected with *C. parvum* oocysts of human and bovine origin, and a *C. felis* infection of a cow has been demonstrated (*15*). These data show not only that the host specificity of some of the *Cryptosporidium* species that infect mammals is less restricted than previously thought but also that there is a complex circulation of species in the environment. Under such circumstances, it is often difficult to trace the source of an infection. In our case, the *C. felis*-infected person did not have a cat at home, but the city where he lives (Rome) is home to a plethora of stray and domestic cats (approximately 0.1 cat per inhabitant). Infection may have occurred upon accidental contact with oocysts in the environment. The public health importance of *Cryptosporidium* parasites other than *C. parvum* needs to be assessed.
